# Supplementary material for: Retrograde and Anterograde Transport of Lat-Vesicles during the Immunological Synapse Formation: Defining the Finely-Tuned Mechanism
Source: Cells. 2021 Feb 9;10(2):359. doi: 10.3390/cells10020359 (PMC7916135; doi:10.3390/cells10020359)
Supplement: Supplementary file 1 [file cells-10-00359-s001.zip › cells-1061100-Supp. Figures -From authors.docx]

Supplementary Material

Retrograde and Anterograde Transport of Lat-Vesicles During the Immunological Synapse Formation: Defining the Finely-Tuned Mechanism


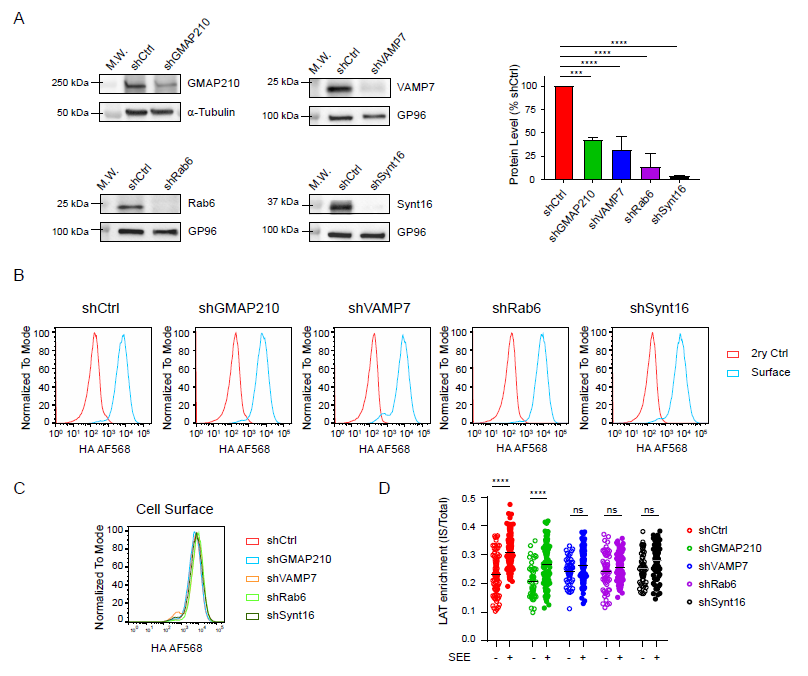


**Figure S1.** GMAP210, VAMP7, Rab6 and Synt16 silencing in Jurkat cells does not affect plasma membrane and total LAT expression. (**A**) Left: Immunoblot showing GMAP210, VAMP7, Rab6 or Synt16 protein levels, together with α-tubulin or GP-96, in the control or corresponding shRNA expressing Jurkat cells lysate. Right: Quantification of GMAP210, VAMP7, Rab6 or Synt16 expression normalized to α -tubulin or GP-96 expression (loading controls) and to control ShRNA, expressed as percentages of the expression of the different proteins in cells expressing the control ShRNA (**B**) Flow cytometry staining of surface HA-LAT (light blue) or total HA-LAT (extracellular staining + intracellular staining; orange) expression in control, GMAP210, VAMP7, Rab6 or Synt16 silenced cells. (**C**) Flow cytometry staining of (**B**) grouped by surface staining in different cells. (**D)** Quantitative analysis of the enrichment of endocytosed LAT at the immune synapse in control or GMAP210/VAMP7/Rab6/Synt16 silenced cells incubated with unpulsed (−, unactivated state) or SEE pulsed (+, immune synapse formation) Raji cells for 30 minutes. One-way ANOVA in A and two-way ANOVA in D. **** *p* < 0.0001; *** *p* < 0.001 ns: non-significant. Data represent three independent experiments.


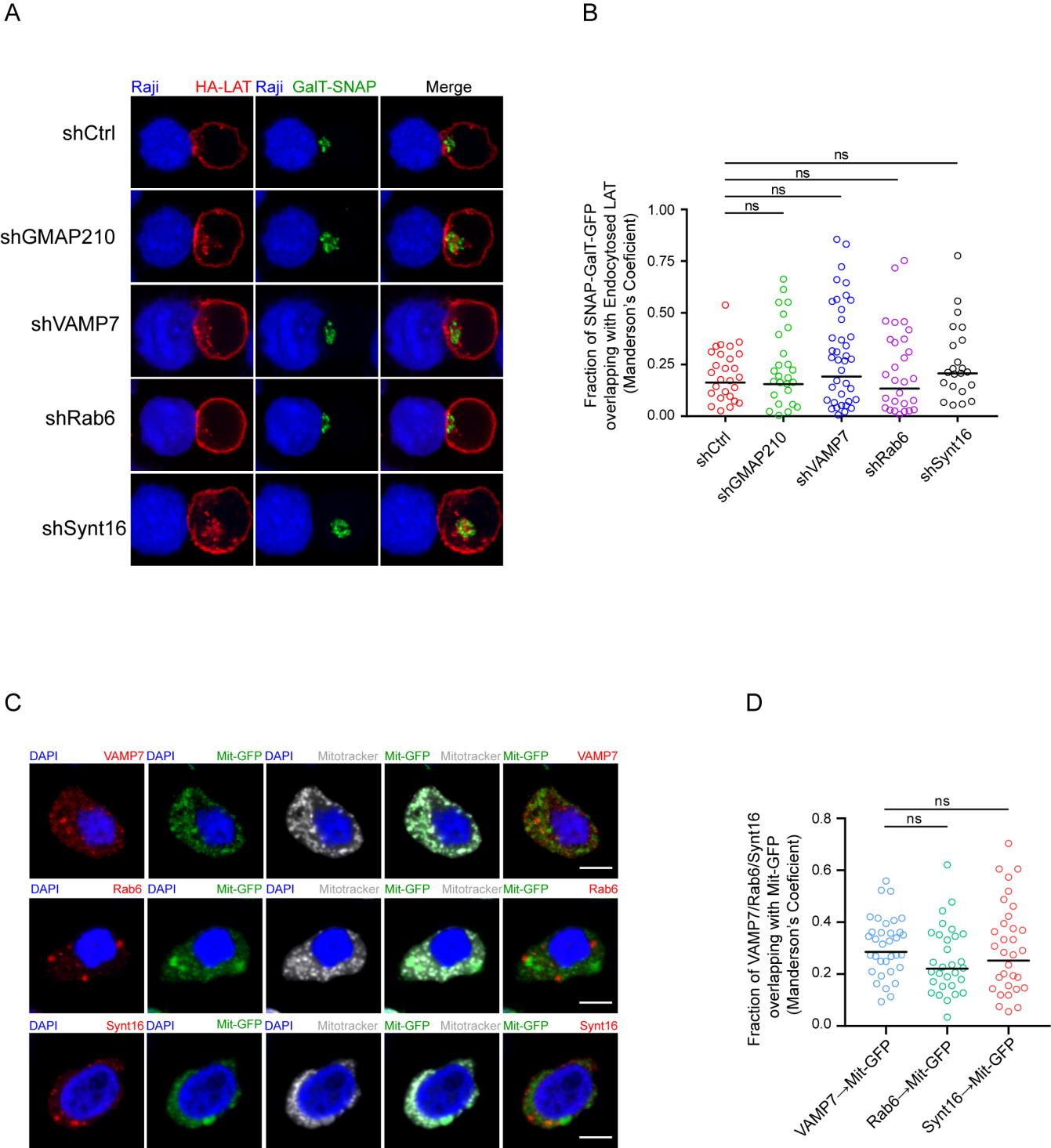


**Figure S2.** Controls for Figure 3. (**A**) Confocal images of Jurkat cells expressing GalT-GFP-SNAP, HA-LAT and control (Ctrl) or GMAP210/VAMP7/Rab6/Synt16 speciﬁc shRNA, stained at 4 °C with anti-HA Ab, washed, and incubated at 4 °C with BG-PEG9-NHS. After washing, cells were activated on slides for 30 min with unpulsed Raji cells unpulsed with SEE (SEE-). Labelings were performed using anti–mouse Ig (Alexa Fluor 568) to label the anti-HA Ab and anti-GFP to label the GalT-GFP-SNAP. Images show the z-projection of summed slices from three stacks covering the Golgi apparatus. Scale bars = 5 μm. (**B**) Quantification of Mander’s overlapping coefficient of GalT-GFP-SNAP with HA-LAT. One-way ANNOVA, ns = non significant. (**C**) Confocal images showing the localization of VAMP7, Rab6 or Synt16 (red) in Jurkat cells expressing a GFP-ActA chimera (GFP-Mit, green), treated for 4 h with nocodazol (nucleus in blue and mitochondria in Gray). (**D**) Quantification of Mander’s overlapping coefficient of GFP-Mit with VAMP7, Rab6 or Synt16. Scale bar 5 μm. Each dot represents one cell; horizontal lines represent the geomean. ns: non-signiﬁcant (Kruskwal-Wallis test). Data and images represent one experiment in (**A**) and (**B**) and two independent experiments in (**C**) and (**D**).
