# Supplementary material for: Retrograde and Anterograde Transport of Lat-Vesicles during the Immunological Synapse Formation: Defining the Finely-Tuned Mechanism
Source: Cells. 2021 Feb 9;10(2):359. doi: 10.3390/cells10020359 (PMC7916135; doi:10.3390/cells10020359)
Supplement: Supplementary file 1 [file cells-10-00359-s001.zip › cells-1061100-Supp. video captions for XML.docx]

Supplementary Material

Retrograde and Anterograde Transport of Lat-Vesicles During the Immunological Synapse Formation: Defining the Finely-Tuned Mechanism

Juan José Saez ^1^, Stephanie Dogniaux ^1^, Massiullah Shafaq-Zadah ^2^, Ludger Johannes ^2^, Claire Hivroz ^1,^* and Andrés Ernesto Zucchetti ^1,^*

**Supplementary Video 1.** Live TIRF imaging of the recruitment of LAT and GMAP210 at the immune synapse. Time-lapse TIRF microscopy images of Jurkat cells co-transfected with LAT-mCherry (red) and GMAP210-GFP (green) seeded on coverslips coated with anti-CD3ε+antiCD28 Abs. Left: LAT-mCherry, middle: GMAP210-GFP, right: merge. Time frame = 5 seconds and Scale bar 5 μm.

**Supplementary Video 2.** Live TIRF imaging of the recruitment of LAT and VAMP7 at the immune synapse. Time-lapse TIRF microscopy images of Jurkat cells co-transfected with LAT-mCherry (red) and VAMP7-GFP (green) seeded on coverslips coated with anti-CD3ε+antiCD28 Abs. Left: LAT-mCherry, middle: VAMP7-GFP, right: merge. Time frame = 5 seconds and Scale bar 5 μm.

**Supplementary Video 3.** Live TIRF imaging of the recruitment of LAT and Rab6A at the immune synapse. Time-lapse TIRF microscopy images of Jurkat cells co-transfected with LAT-mCherry (red) and Rab6A-GFP (green) seeded on coverslips coated with anti-CD3ε+antiCD28 Abs. Left: LAT-mCherry, middle: Rab6A-GFP, right: merge. Time frame = 5 seconds and Scale bar 5 μm.
